# The age-specific differences in histopathological tumor characteristics and TNM classification of breast carcinomas in Quality assured mamma diagnostic (QuaMaDi) program in the state of Schleswig–Holstein in Germany

**DOI:** 10.1007/s00432-021-03841-x

**Published:** 2021-10-27

**Authors:** L.-J. Kramp, M. Mathiak, H.-M. Behrens, F. W. Schäfer, M. van Mackelenbergh, Christoph Röcken

**Affiliations:** 1 Department of Pathology, University Hospital Schleswig-Holstein, Campus Kiel, Arnold-Heller-Str. 3, Haus U33, 24105 Kiel, Germany; 2 Department of Gynecology and Obstetrics, University Hospital Schleswig-Holstein, Campus Kiel, Kiel, Germany

**Keywords:** Breast cancer, Quality assurance, Mammography, Immunohistochemistry, Molecular subtype, Age

## Abstract

**Background:**

We explored the hypothesis that high-quality standards in diagnostic mammography can lead to an early diagnosis of breast cancers and identifies at risk populations outside screening programs. The histopathological features and distribution of the TNM classification were examined in relation to patient age in a large group of women with breast cancers participating in the Quality Assured Mamma Diagnostic (QuaMaDi) program of the state of Schleswig–Holstein.

**Patients and methods:**

Surgical pathological reports were studied for clinicopathological characteristics, receptor status, molecular subtype and tumor stage. The analysis was conducted by dividing the study population into three age groups: women under 50 years (pre-screening), 50–69 years (peri-screening) and over 70 years (post-screening).

**Results:**

7.111 biopsies and 2.887 resection specimens were included. Breast cancer was diagnosed in 4.241 (59.7%) cases, one fourth of them in women < 50 years. Elderly women (> 70 years) had more well-differentiated, estrogen receptor (ER)-positive and HER2-negative carcinomas, whereas younger women (< 50 years) tended to have more poorly differentiated, ER negative, and HER2-positive carcinomas. 47% of breast carcinoma were luminal B tumors and were most common regardless of age. 70.4% of resected specimen had pT1 stage. Nodal negative were 71.2%.

**Conclusion:**

In QuaMaDi breast cancer was diagnosed at an early and potentially curable stage of the disease due to high-quality standards in diagnostic mammography. In addition, regardless of age, an increased number of prognostically unfavorable molecular subtypes were detected. Thus, QuaMaDi helps to identify at risk populations. QuaMaDi significantly improves diagnostic mammography and complements mammography screening programs.

**Supplementary Information:**

The online version contains supplementary material available at 10.1007/s00432-021-03841-x.

## Introduction

Female breast cancer is the most commonly diagnosed cancer in the world (Global Cancer Observatory 2020). Over the past decades, immense efforts have been made to improve early diagnosis and treatment. Screening programs using mammography aim to identify asymptomatic cancer at an early disease stage, thereby reducing cancer mortality and improving life quality (Andersson et al. 1988; Andersson and Janzon 1997; Bjurstam et al. 2003; Roberts et al. 1990; Sardanelli et al. 2017). Recent European guidelines recommend the extension of mammography screening to a greater age group (European Commission Initiative on Breast Cancer 2019a, b). Nevertheless, these screening programs are not available to all women. In Germany, asymptomatic women aged between 50 and 69 years are offered a mammogram biannually (Robert Koch Institute 2016). However, women outside this age range or with breast cancer-related symptoms are excluded from the German Mammography Screening Program (GMSP) and obtain diagnostics in standard care (Institute for Cancer Epidemiology 2006). This indication-based mammography outside GMSP is not regulated by high-quality standards implemented by the GMSP (Institute for Cancer Epidemiology 2006). Symptomatic breast cancer, often associated with a diagnostic delay, is usually associated with more aggressive tumor characteristics and therefore with a higher mortality rate than screening-detected breast cancer. In Germany, approximately 53% of incidental breast cancer cases are diagnosed outside the GMSP (Robert Koch Institute and the Association of Population-based Cancer Registries in Germany 2019). Furthermore, half of women between 50 and 69 years do not participate in the GMSP (Cooperative Association of the German Mammography Screening Program 2019).

In Schleswig–Holstein (SH), the most northern federal state in Germany, the pilot-project: Quality Assured Mamma Diagnostic (QuaMaDi) was initiated in 1999 to improve the quality of indication-based breast diagnostics. QuaMaDi offers a standardized, evaluated diagnostic process including a clinical examination and an independent double reporting of mammography by two radiologists. Women who either are at risk for breast cancer, have symptoms or already have had breast cancer (after-treatment care) can participate in QuaMaDi regardless of age. If necessary, further non-invasive or invasive diagnostics (e.g., biopsy) is provided by the reference center (certified breast center) (Obi et al. 2011). Quality-standardized documentation is carried out. Details of QuaMaDi have been described elsewhere (Obi et al. 2011; Katalinic et al. 2007; Schaefer et al. 2010). Although QuaMaDi is not a screening program, it aims to identify breast cancer at an early disease stage (Institute for Cancer Epidemiology 2006). Due to excellent results within the pilot region between 2001 and 2005 QuaMaDi has been implemented in standard care in SH (Katalinic et al. 2007). Until now, no study has systematically reviewed the biopsy and resection specimens obtained within QuaMaDi. Therefore, we carried out a retrospective, single center study on all women who had participated in QuaMaDi between 2005 and 2016 at the University Hospital Schleswig–Holstein. We aimed to test the following hypothesis: (1) QuaMaDi identifies at risk populations at time; (2) it is a highly valuable additive to GMSP; and (3) biopsy specimens obtained during QuaMaDi are representative for breast lesions, which were forwarded to surgical and/or oncological treatment.

## Patients and methods

### Ethics statement

Our project was granted ethical clearance by the local ethics committee of the University Hospital in Kiel, Germany, in agreement with the Helsinki Declaration (D470/17).

### Study population

From the electronic database of the Department of Pathology, University Hospital Schleswig–Holstein, Campus Kiel, we retrieved all patients, who were part of QuaMaDi between 01.01.2005 and 31.12.2016. Only women were included if (1) the referral indicated “QuaMaDi” and (2) a biopsy specimen was submitted to the Dept. of Pathology. Patients were excluded if they were (1) of male gender or (2) if only cytological specimens were submitted. The following patient characteristics were retrieved from the electronic database: age, gender, date of biopsy, biopsy site, number of biopsy samples, histological diagnoses, and the B-classification (since 2007). The following additional data were retrieved for non-invasive and invasive cancers: histological type of the neoplastic lesion, anatomical site, immunoreactivity score (IRS) for the estrogen (ER) and progesterone receptor (PR) (in-situ lesions and invasive cancer), and additionally Elston and Ellis grading (since 2009), HER2 status and Ki67-index (since 2012) for invasive cancers. If patients had undergone surgery at the Dept. of Gynecology and Obstetrics, University Hospital Kiel, the following data were retrieved: date of surgery, age of the patient at the time of surgery, adjuvant or neoadjuvant therapy regimen, histological tumor type, pTNM-classification, grading, and tumor regression (according to Sinn regression score, Sinn et al. 1994). All patient-related data were pseudonymized after inclusion into the study.

### Histology

Tissue specimens were fixed in formalin and embedded in paraffin. Deparaffinized sections were stained with hematoxylin and eosin. The grading system of Elston and Ellis was applied since 2009 to invasive cancers (Elston and Ellis 1991). The pTNM-stage of all study patients was determined according to sixth and seventh editions of the UICC guidelines. All biopsy and resection specimens had been examined by trained and board-certified surgical pathologists.

### B-classification

According to national guidelines for the diagnosis and treatment of breast cancer, the histopathological lesions in core (NCB) and vacuum-assisted biopsies (VAB) were categorized according to the B-classification (Ellis et al. 2004). In brief: category B1 denotes normal breast tissue or insufficient sampling, B2 benign lesions, B3 benign lesions of unknown biological potential, B4 lesions suspicious for malignancy, B5a in situ carcinomas, B5b invasive breast cancer, B5c indiscernible whether non-invasive or invasive, and B5d malignant tumor of divergent histology (e.g., malignant phylloides tumor) (S1).

### Immunohistochemistry and in situ hybridization

Immunohistochemical staining was done using a Bondmax automated slide staining system (Leica Biosystems, Wetzlar, Germany), the Polymer Refine Detection Kit (Leica Biosystems) and antibodies directed against ER (dilution 1:150), PR (both Leica Biosystems Newcastle, Newcastle, United Kingdom; dilution 1:100), Her2/neu (1:100) and anti-Ki-67 antibody (both Thermo Fisher Scientific, Fermont, USA; dilution 1:300). For chromogenic in situ hybridization the ZytoDot^®^ 2C SPEC ERB2/CEN 17 Probe Kit (ZytoVision GmbH, Bremerhaven, Germany) was used.

### Scoring and assessment of estrogen and progesterone receptor status

The ER and PR status were assessed according to Remmele and Stegner (1987). In brief: Category A documented the intensity of nuclear staining as absent (0), weak (1), moderate (2) or strong (3). Category B documented the percentage of stained tumor cells as absent (0), < 10% (1), 10–50% (2), 51–80% (2), and > 80% positive nuclei (4). The immunoreactivity score (IRS) was calculated according to the formula: Category A (immunostaining intensity) × Category B (proportion of positive cells of the tumor). An IRS of 0 and 1 was interpreted as receptor negative. IRS ≥ 2 were considered receptor positive. The range of 2–3 represented a weak, 4–8 a moderately strong, and 9–12 a strong expression of the hormone receptors. Over time the IRS was abandoned, because scores between 1 and 3 could indicate both receptor negative and positive status. Hence, the percentage of positive tumor cells in the specimen was crucial for the assessment. Since 2010, each tumor was assessed according to ASCO/CAP guidelines for ER and PR testing (Hammond et al. 2010). Hormone receptor status for ER and PR was considered positive if ≥ 1% of tumor cells were immunoreactive. Breast carcinoma was hormone receptor negative if < 1% of tumor cells stained (with positive internal control). The IRS was still optionally reported in the pathological findings. Finally, the hormone receptor status was determined by using the documented IRS in the pathological report. Since an IRS of 1, 2 and 3 could indicate both a negative and positive hormone receptor status, assignment to the categories hormone receptor positive and negative was based on the pathologist’s evaluation as documented in the pathology report. Ambiguous reports were re-examined during this study by a board-certified surgical pathologist. All IRS values ≥ 4 were assigned to positive hormone receptor status.

### Scoring and assessment of Ki-67

Since 2012, the Ki-67 index was assessed according to recommendations of the International Ki-67 in Breast Cancer Working Group (Dowsett et al. 2011). After prior overview of the entire tissue specimen, the evaluation of the Ki-67 proliferation index was carried out using at least 3 high-power (× 40 objective) fields. The fields were selected to reflect the staining of the whole specimen. The invasion front of the tumor was evaluated. Only nuclear staining was assessed. The Ki-67 proliferation index is defined as the percentage of positively stained cells among the total number of malignant cells scored. A minimum of 100 cells was analyzed and determined by single cell counting using a counting device. If distinct clusters of positively stained cells were seen in the preparation, they were included in the overall evaluation. Finally, the Ki-67 index was categorized into the following groups: ≤ 10%, 10–20%, 20–25%, 25–50%, > 50% and unclassifiable. Ranges and no exact value of Ki-67 were assigned as unclassifiable.

### Scoring and assessment of HER2 status

Scoring of each breast carcinoma was assessed on core biopsy specimens according to the ASCO/CAP recommendations for HER2 testing (Wolff et al. 2007, 2013). The immunostaining intensity and extent as well as percentage of positive tumor cells was evaluated. On slide positive controls were used in each case. During study period, the recommendations of ASCO/CAP were constantly updated.

### Assessment of chromogenic in situ hybridization

If the HER2 Score was 2 + a reflex test on the same biopsy specimen was performed using the chromogenic in situ hybridization according to the ASCO/CAP recommendations for HER2 testing (Wolff et al. 2007, 2013). The amplification was determined by examining average *HER2* copy number and since 2007 the *HER2*/CEP 17 ratio. During study period, the recommendations of ASCO/CAP were constantly updated. The in situ hybridization status used in this study are listed.

### Molecular subtypes

The classification of the molecular subtypes was based on the St. Gallen recommendations (Goldhirsch et al. 2011) with IHC analysis of ER, PR, HER2, Ki-67 and CISH analysis of *HER2*. The molecular subtypes were defined as follows: luminal A (ER positive, HER2 negative, Ki-67 < 20%), luminal B (either ER positive, HER2 positive and Ki-67 ≥ 20% or ER positive, HER2 negative and Ki-67 ≥ 20%), HER2 positive (ER negative, HER2 positive, any Ki-67) and triple-negative (ER negative, HER2 negative, any Ki-67).

### Internal and external quality assurance schemes

During the entire study period, the Dept. of Pathology regularly participated successfully in external quality assurance schemes. These comprised the annual participation in the Quality Assurance Initiative for Pathology (QuIP) for ER, PR, Ki-67, HER2, and *HER2* CISH. The proficiency test results are published and available on the homepage of the Dept. of Pathology (https://www.patho.uni-kiel.de/krankenversorgung/qualitätssicherung). In addition, the Dept. of Pathology voluntarily participated in the HER2-Monitor of the Hannover Medical School. There, the internal positivity rate of HER2 is constantly compared with the nationwide average. Furthermore, the histological examinations were carried out by trained and board-certified surgical pathologists and any ambiguous finding was re-assessed by at least one additional board-certified surgical pathologist (four-eye principle).

### Statistics

SPSS version 25.0.0.2 (IBM Corp., Armonk, NY, USA) was used for statistical analyses. To test for correlation between non-ordinal variables, we used Fisher´s exact test. When testing for correlation between variables of nominal scale, we used Pearson´s chi square test. We assumed a significance level of 0.05. The *p*-values were calculated by two-sided *T*-tests and a *p*-value ≤ 0.01 was considered highly significant.

## Results

### Characteristics of the study population

In total, 7.111 biopsy specimens and 2.887 corresponding resection specimens obtained from 6.845 women were assigned to the QuaMaDi program. The median age of the patients was 55 years (range from 15 to 94 years for biopsy specimens; 26 to 94 years for resection specimens). 6.586 women participated in the QuaMaDi program once, 252 twice and 7 three times. In 782 (11.4%) women, more than one biopsy (different localization within the breast or different anatomical sites) was obtained per referral. We categorized our patients into three age groups: pre- (< 50 years), peri- (50–69 years) and post-screening group (≥ 70 years): 2.685 (37.7%) of 7.111 histological referrals were obtained from women < 50 years (median age 44 years; age range 15–49), 2.935 (41.3%) from women between 50 and 69 years (median age 60; age range 50–69), and 1.491 (21.0%) from women ≥ 70 years (median age 75; age range 70–94).

### Comparison of QuaMaDi patients with a single biopsy

First, we correlated the three different age groups with various pathological patient characteristics, i.e., anatomical localization (C-code), B-category, tumor type (M-category), the presence of precursor lesions, tumor grade, receptor status and Ki-67 index and molecular subtypes in patients with a single biopsy. As summarized in Table 1 significant differences between the three age groups were found for all parameters except the Ki-67 index.

**Table 1:** Comparison of histological submissions, which contain one biopsy (= 1 biopsy), based on the age groups pre-, peri- and post-screening

		Total		Age group						*p*-value
				Pre-screening (< 50 years)		Peri-screening (50–69 years)		Post-screening (≥ 70 years)		
		*n* (%)		*n* (%)		*n* (%)		*n* (%)		
Localization	Total number [*n*]	6.329								0.007 ^a^
C50.1		109 (1,7)		34 (1.4)		39 (1.5)		36 (2.7)		
C50.2		383 (6.1)		149 (6.2)		162 (6.2)		72 (5.4)		
C50.3		205 (3.2)		80 (3.3)		76 (2.9)		49 (3.7)		
C50.4		1.377 (21.8)		530 (22.1)		586 (22.5)		261 (19.7)		
C50.5		305 (4.8)		128 (5.3)		120 (4.6)		57 (4.3)		
C50.6		36 (0.6)		5 (0.2)		22 (0.8)		9 (0.7)		
C50.8		1.197 (18.9)		463 (19.3)		493 (18.9)		241 (18.2)		
C50.9		2.717 (42.9)		1.008 (42.1)		1.110 (42.6)		599 (45.2)		
B-classification	Total number [*n*]	4.433								0.000^a^
B1		151 (3.4)		78 (4.5)		55 (3.3)		18 (1.7)		
B2		1.408 (31.8)		879 (50.5)		406 (24.7)		123 (11.7)		
B3		283 (6.4)		151 (8.7)		93 (5.6)		39 (3.7)		
B4		53 (1.2)		12 (0.7)		24 (1.5)		17 (1.6)		
B5a		260 (5.9)		73 (4.2)		123 (7.5)		64 (6.1)		
B5b		2.232 (50.3)		536 (30.8)		930 (56.5)		766 (73.2)		
B5c		46 (1.0)		10 (0.6)		16 (1.0)		20 (1.9)		
B5d		0 (0.0)		0 (0.0)		0 (0.0)		0 (0.0)		
Carcinoma	Total number [*n*]	3.084								
Invasive ductal carcinoma/ invasive carcinoma NST		2.318 (75.2)		573 (81.3)		1.062 (75.4)		683 (70.3)		0.000^b^
Invasive lobular carcinoma		486 (15.8)		82 (11.6)		234 (16.6)		170 (17.5)		0.000 ^b^
Invasive carcinoma with medullary features/ medullary carcinoma/atypical medullary carcinoma		59 (1.9)		24 (3.4)		22 (1.6)		13 (1.3)		0.175 ^b^
Other invasive carcinomas		221 (7.1)		26 (3.7)		90 (6.4)		105 (10.9)		
Tubular carcinoma		50 (1.6)		8 (1.1)		27 (1.9)		15 (1.5)		0.004^b^
Invasive papillary carcinoma/solid papillary carcinoma		36 (1.2)		4 (0.6)		12 (0.9)		20 (2.1)		0.005^b^
Metaplastic carcinoma		6 (0.2)		0 (0.0)		2 (0.1)		4 (0.4)		0.135^b^
Mucinous carcinoma		65 (2.1)		7 (1.0)		17 (1.2)		41 (4.2)		0.000^b^
Other subtypes		36 (1.2)		5 (0.7)		16 (1.1)		15 (1.5)		0.046^b^
Metastases		18 (0.6)		1 (0.1)		12 (0.9)		5 (0.5)		0.006^b^
Other malignant tumors		9 (0.3)		1 (0.1)		4 (0.3)		4 (0.4)		0.368^b^
Multiple malignant carcinomas		1 (0.0)		0 (0.0)		0 (0.0)		1 (0.1)		0.386^b^
In-situ lesions	Total number [*n*]	529								
Atypical epithelial proliferation of ductal type/ DCIS non high grade /ADH		300 (56.7)		85 (55.2)		150 (56.6)		65 (59.1)		0.000^b^
Ductales carcinoma in situ high grade		171 (32.3)		49 (31.8)		83 (31.3)		39 (35.5)		0.000^b^
Lobular intraepithelial neoplasia		16 (3.0)		4 (2.6)		11 (4.2)		1 (0.9)		0.007^b^
Flat epithelial Atypia		23 (4.3)		13 (8.4)		9 (3.4)		1 (0.9)		0.008^b^
Multiple in-situ carcinoma		19 (3.6)		3 (1.9)		12 (4.5)		4 (3.6)		0.021^b^
Elston & Ellis grading system	Total number [*n*]	1.602								0.025^a^
G1		463 (28.9)		96 (25.3)		186 (28.9)		181 (31.3)		
G2		899 (56.1)		212 (55.8)		358 (55.6)		329 (56.9)		
G3		240 (15.0)		72 (18.9)		100 (15.5)		68 (11.8)		
Estrogen receptor status	Total number [*n*]	3.503								0.000^a^
Negative		506 (14.4)		150 (18.3)		229 (14.1)		127 (11.9)		
Positive (weak expression)		145 (4.1)		45 (5.5)		64 (4.0)		36 (3.4)		
Positive (moderate expression)		661 (18.9)		228 (27.9)		286 (17.7)		147 (13.8)		
Positive (strong expression)		2.191 (62.5)		395 (48.3)		1.040 (64.2)		756 (70.9)		
Progesterone receptor status	Total number [*n*]	3.497								0.099^a^
Negative		950 (27.2)		226 (27.7)		448 (27.7)		276 (25.9)		
Positive (weak expression)		237 (6.8)		43 (5.3)		123 (7.6)		71 (6.7)		
Positive (moderate expression)		838 (24.0)		178 (21.8)		395 (24.4)		265 (24.9)		
Positive (strong expression)		1.472 (42.1)		369 (45.2)		651 (40.3)		452 (42.5)		
HER2 status	Total number [*n*]	3.068								0.000^a^
Negative		2.656 (86.6)		585 (83.1)		1.189 (84.9)		882 (91.6)		
Positive		412 (13.4)		119 (16.9)		212 (15.1)		81 (8.4)		
Positive (% within HER2-Status)				119 (28.9)		212 (51.5)		81 (19.7)		
Ki-67-index (grouped)	Total number [*n*]	927								0.160^a^
≤ 10		110 (11.9)		22 (10.0)		41 (11.3)		47 (13.7)		
10–20		219 (23.6)		43 (19.5)		83 (22.9)		93 (27.1)		
20–25		105 (11.3)		25 (11.3)		42 (11.6)		38 (11.1)		
25–50		299 (32.3)		75 (33.9)		119 (32.8)		105 (30.6)		
> 50		153 (16.5)		48 (21.7)		63 (17.4)		42 (12.2)		
Unclassifiable		41 (4.4)		8 (3.6)		15 (4.1)		18 (5.2)		
IHC based molecular subtypes	Total number [*n*]	1.322								0.000^a^
Triple-negative (ER- PR- HER2-)		301 (22.8)		99 (28.2)		123 (22.1)		79 (19.1)		
Luminal A		297 (22.5)		60 (17.1)		108 (19.4)		129 (31.2)		
Luminal B		622 (47.0)		160 (45.6)		277 (49.7)		185	(44.7)	
HER2 positive		102 (7.7)		32 (9.1)		49 (8.8)		21 (5.1)		

^a^Fishers exact test

^b^Chi square tes

The percentage of invasive tumors (B5b) increased from 30.8% in the pre- to 73.2% in the post-screening group, while the percentage of benign lesions (B2) decreased from 50.5 to 11.7%. The proportion of benign lesions with uncertain biological potential (B3) halved from 8.3 to 3.7% (Table 1). While the risk of detecting cancer increased continuously with age, a substantial number of women aged < 50 years, i.e., 35% had an in-situ carcinoma (B5a) or an invasive carcinoma (B5b).

Invasive carcinoma NST was found most commonly in the pre-screening group (81.3%), while invasive lobular (17.5%) and mucinous (4.2%) carcinomas were found most commonly in the post-screening group (Table 1). Conclusively, although invasive carcinoma NST was the most common tumor type in each age group, better differentiated carcinomas, such as mucinous carcinomas, were more likely to occur with increasing age.

Likewise, the detection rate of precursor lesions increased significantly with patient age (Table 1). 29% of the high-risk lesions and in situ carcinomas occurred in women < 50 years. The proportion of well-differentiated carcinomas (G1) also increased with age, whereas the proportion of poorly differentiated carcinomas (G3) decreased significantly from 18.9% to 11.8% (Table 1). Regardless of age, moderately differentiated carcinoma (G2) was diagnosed in more than half of the cases. The results show that younger women (< 50 years) were more likely to have more aggressive, poorly differentiated carcinomas.

There were highly significant differences for the ER- and HER2 status: The proportion of ER-positive carcinomas increased from 81.7% in the pre- to 88.1% in the post-screening group. The percentage of HER2-positive carcinomas halved from 16.9 to 8.4%. No significant differences between the three age groups were demonstrated for the PR status and Ki-67 index. Overall, the results indicate that elderly women (≥ 70 years) show a higher frequency of ER positive and HER2-negative carcinomas, whereas those under 50 years tended to have ER negative and HER2-positive carcinomas, underscoring the higher frequency of more aggressive breast carcinomas in the younger age group.

The significant differences in the hormone receptor status mirrored in the four molecular subtypes, i.e., luminal A, luminal B, HER2 positive and triple-negative. The proportion of triple-negative breast carcinomas decreased significantly from 28.2% in the pre- to 19.1% in the post-screening group (Table 1). In contrast, the proportion of Luminal A carcinomas doubled from 17.1% in the pre- to 31.2% in post-screening group. Luminal B occurred most frequently in all age groups, accounting for more than 40%. Overall, women < 50 years exhibited 30.5% triple-negative and HER2-positive carcinomas.

### Detection frequency of malignancy in the entire cohort

**Fig. 1:**
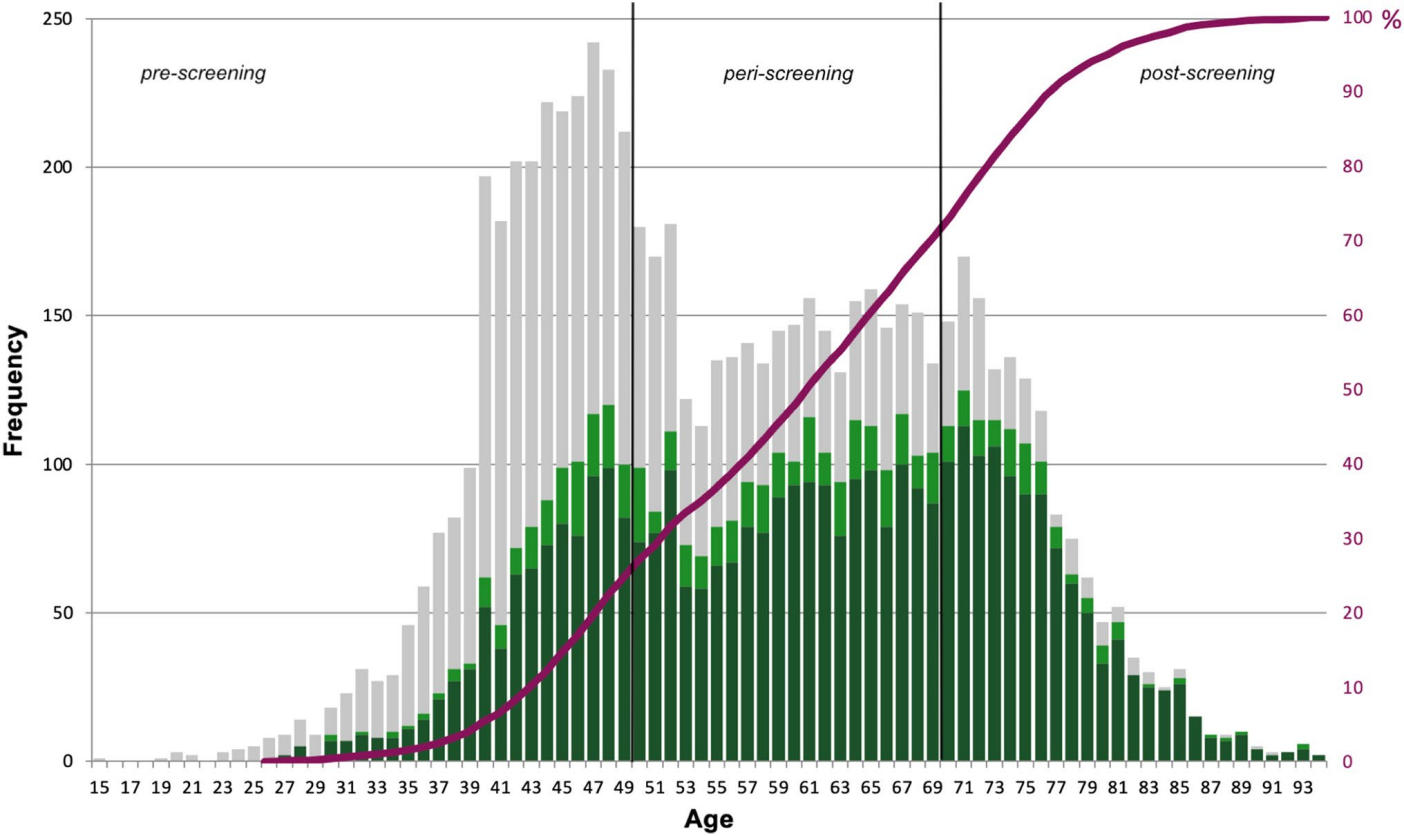
Absolute age distribution of 7.111 histological submissions at the time of diagnosis in the period from 01/01/2005 to 31/12/2016, color-divided by the presence or absence of malignancy. The black vertical lines mark the age groups pre-, peri-, and post-screening. The red curve describes the cumulative proportion of the incidence of invasive breast carcinoma and precursor lesions in relation to age at diagnosis

A total of 7.111 clarification diagnoses consisting of both single and multiple biopsies were categorized into the three age groups. The comparison of the three age groups showed a highly significant difference. The proportion of invasive carcinomas increased from 32.6% in the pre-screening to 74.6% in the post-screening group (S2). In particular, a linear increase of malignant breast lesions of 2.4% per year of age was observed in the age range between 43 and 77 years (Fig. 1). In addition, the ratio of benign to malignant lesions increased from an initial 1:0.64 for women < 50 years to 1:1.99 for women between 50 and 69 years. A markedly higher ratio of benign to malignant lesions, i.e., 1:4.87, occurred for women ≥ 70 years. Overall, 54.5% of breast carcinomas appeared beyond the screening age. In particular, 23.9% of the diagnosed carcinomas were detected in women < 50 years.

### Resection specimens in patients who took part in QuaMaDi

We identified 2.887 resection specimens of QuaMaDi, of which 2.454 cases had been assigned to primary surgical therapy (non-neo group) and 433 had received neoadjuvant therapy (neo group) (Table 2). Comparing the three age groups, we found significant differences regarding tumor type, local tumor growth (pT category), nodal spread (pN category), lymph vessel invasion (pL category), tumor grade and resection status (R category) in patients who were primarily resected and had not received neoadjuvant treatment (Table 2). While in women of the neo group, significant differences were only found for the pT category. In all three age groups, the tumor stage pT1c was found most commonly in the resection specimens of the non-neo group. Overall, 70.3% of the untreated carcinomas in the pre-, 75.2% in the peri- and 63.3% in the post-screening group had a tumor size < 2 cm (pT1). In the neoadjuvant treatment regimen, a pathologic complete remission (defined as the absence of tumor residuals in the breast and axilla) was achieved in one-fifth of the patients in both the pre- and peri-screening groups (ypT0). These results show that the majority of the carcinomas were detected at an early, more prognostically favorable stage of the disease in all three age groups. However, higher and prognostically less favorable tumor stages were found with a higher probability in women < 50 and ≥ 70 years.

**Table 2:** Evaluation of the resection specimens comparing the three age groups pre-, peri- and post-screening, divided by the used treatment concept

	Total		Total no neoadjuvant therapy		No neoadjuvante therapy						*p*-value	Total neoadjuvant therapy			Neoadjuvant therapy						*p*-value
					Pre-screening (< 50 years)		Peri-screening (50–69 years)		Post-screening (≥ 70 years)		Fisher´s exact test				Pre-screening (< 50 years)		Peri-screening (50–69 years)		Post-screening (≥ 70 years)		Fisher´s exact test
	*n *(%)		*n *(%)		*n *(%)		*n *(%)		*n *(%)			*n *(%)			*n *(%)		*n *(%)		*n *(%)		
ICD-M (grouped)	2.886		2.453								0.000	433									0.062
Invasive carcinoma not specified	1.886 (65.3)		1.569 (64.0)		330 (67.2)		760 (64.4)		479 (61.3)			317 (73.2)			161 (74.9)		127 (70.2)		29 (78.4)		
Lobular carcinoma not specified	425 (14.7)		358 (14.6)		49 (10.0)		167 (14.1)		142 (18.2)			67 (15.5)			28 (13.0)		36 (19.9)		3 (8.1)		
Medullary carcinoma not specified. atypical medullary carcinoma	56 (1.9)		40 (1.6)		15 (3.1)		15 (1.3)		10 (1.3)			16 (3.7)			9 (4.2)		7 (3.9)		0 (0.0)		
Tubular adenocarcinoma	42 (1.5)		39 (1.6)		7 (1.4)		22 (1.9)		10 (1.3)			3 (0.7)			3 (1.4)		0 (0.0)		0 (0.0)		
Intraductal papillary adenocarcinoma with invasion	17 (0.6)		16 (0.7)		1 (0.2)		7 (0.6)		8 (1.0)			1 (0.2)			0 (0.0)		0 (0.0)		1 (2.7)		
Metaplastic carcinoma not specified	10 (0.3)		9 (0.4)		1 (0.2)		3 (0.3)		5 (0.6)			1 (0.2)			0 (0.0)		1 (0.6)		0 (0.0)		
Mucinous carcinoma	44 (1.5)		41 (1.7)		1 (0.2)		11 (0.9)		29 (3.7)			3 (0.7)			2 (0.9)		1 (0.6)		0 (0.0)		
Other subtypes	90 (3.1)		81 (3.3)		9 (1.8)		37 (3.1)		35 (4.5)			9 (2.1)			2 (0.9)		4 (2.2)		3 (8.1)		
In-situ carcinoma	316 (10.9)		300 (12.2)		78 (15.9)		159 (13.5)		63 (8.1)			16 (3.7)			10 (4.7)		5 (2.8)		1 (2.7)		
pT category	2.887		2.454								0.000	433									0.037
T0	81 (2.8)		0 (0.0)		0 (0.0)		0 (0.0)		0 (0.0)			81 (18.7)			43 (20.0)		35 (19.3)		3 (8.1)		
Tis	321 (11.1)		301 (12.3)		77 (15.7)		160 (13.6)		64 (8.2)			20 (4.6)			12 (5.6)		6 (3.3)		2 (5.4)		
T1mi	15 (0.5)		13 (0.5)		4 (0.8)		7 (0.6)		2 (0.3)			2 (0.5)			1 (0.5)		1 (0.6)		0 (0.0)		
T1a	112 (3.9)		81 (3.3)		19 (3.9)		36 (3.0)		26 (3.3)			31 (7.2)			21 (9.8)		10 (5.5)		0 (0.0)		
T1b	407 (14.1)		375 (15.3)		70 (14.3)		203 (17.2)		102 (13.0)			32 (7.4)			21 (9.8)		9 (5.0)		2 (5.4)		
T1c	1.038 (36.0)		958 (39.0)		175 (35.6)		482 (40.8)		301 (38.5)			80 (18.5)			41 (19.1)		30 (16.6)		9 (24.3)		
T2	726 (25.2)		612 (24.9)		125 (25.5)		257 (21.8)		230 (29.4)			114 (26.3)			51 (23.7)		51 (27.6)		13 (35.1)		
T3	164 (5.7)		98 (4.0)		21 (4.3)		32 (2.7)		45 (5.8)			66 (15.2)			24 (11.2)		36 (19.9)		6 (16.2)		
T4a	1 (0.0)		0 (0.0)		0 (0.0)		0 (0.0)		0 (0.0)			1 (0.2)			0 (0.0)		1 (0.6)		0 (0.0)		
T4b	13 (0.5)		9 (0.4)		0 (0.0)		2 (0.2)		7 (0.9)			4 (0.9)			1 (0.5)		2 (1.1)		1 (2.7)		
T4d	5 (0.2)		4 (0.2)		0 (0.0)		0 (0.0)		4 (0.5)			1 (0.2)			0 (0.0)		1 (0.6)		0 (0.0)		
TX	4 (0.1)		3 (0.1)		0 (0.0)		2 (0.2)		1 (0.1)			1 (0.2)			0 (0.0)		0 (0.0)		1 (2.7)		
rpT-category	2.885		2.453								0.068	432									0.001
Relapse	92 (3.2)		89 (3.6)		10 (2.0)		44 (3.7)		35 (4.5)			3 (0.7)			0 (0.0)		0 (0.0)		3 (8.3)		
No relapse	2.793 (96.8)		2.364 (96.4)		481 (98.0)		1136 (96.3)		747 (95.5)			429 (99.3)			215 (100)		181 (100)		33 (91.7)		
pT(m)-category	2.566		2.153								0.489	413									0.966
Multiple lesions	320 (12.5)		283 (13.1)		57 (13.8)		125 (12.2)		101 (14.1)			37 (9.0)			19 (9.4)		15 (8.6)		3 (8.6)		
No multiple lesions	2.246 (87.5)		1.870 (86.9)		357 (86.2)		896 (87.8)		617 (85.9)			376 (91.0)			184 (90.6)		160 (91.4)		32 (91.4)		
pN category	2.665		2.239								0.012	426									0.171
N0	1.871 (70.2)		1.607 (71.8)		305 (68.7)		799 (74.5)		502 (69.7)			264 (62.0)			139 (65.3)		105 (59.7)		20 (54.1)		
N1mi	73 (2.7)		60 (2.7)		18 (4.1)		23 (2.1)		19 (2.6)			13 (3.1)			8 (3.8)		5 (2.8)		0 (0.0)		
N1a	421 (15.8)		339 (15.1)		77 (17.3)		154 (14.4)		108 (15.0)			82 (19.2)			42 (19.7)		31 (17.6)		9 (24.3)		
N1b	1 (0.0)		1 (0.0)		0 (0.0)		0 (0.0)		1 (0.1)			0 (0.0)			0 (0.0)		0 (0.0)		1 (2.5)		
N1c	2 (0.1)		2 (0.1)		0 (0.0)		0 (0.0)		2 (0.3)			0 (0.0)			0 (0.0)		0 (0.0)		0 (0.0)		
N2a	142 (5.3)		97 (4.3)		23 (5.2)		45 (4.2)		29 (4.0)			45 (10.6)			19 (8.9)		22 (12.5)		4 (10.8)		
N3a	77 (2.9)		58 (2.6)		10 (2.3)		18 (1.7)		30 (4.2)			19 (4.5)			5 (2.3)		11 (6.3)		3 (8.1)		
NX	78 (2.9)		75 (3.3)		11 (2.5)		34 (3.2)		30 (4.2)			3 (0.7)			0 (0.0)		2 (1.1)		1 (2.7)		
pL category	2.563		2.165								0.002	398									0.118
L0	2.207 (86.1)		1.887 (87.2)		346 (82.8)		924 (89.6)		617 (86.2)			320 (80.4)			155 (78.7)		141 (84.4)		24 (70.6)		
L1	353 (13.8)		275 (12.7)		72 (17.2)		106 (10.3)		97 (13.5)			78 (19.6)			42 (21.3)		26 (15.6)		10 (29.4)		
LX	3 (0.1)		3 (0.1)		0 (0.0)		1 (0.1)		2 (0.3)			0 (0.0)			0 (0.0)		0 (0.0)		0 (0.0)		
pV-categroy	2.563		2.164								0.013	399									1.000
V0	2.525 (98.5)		2.137 (98.8)		413 (99.0)		1.024 (99.3)		700 (97.8)			388 (97.2)			193 (97.5)		162 (97.0)		33 (97.1)		
V1	37 (1.4)		26 (1.2)		4 (1.0)		6 (0.6)		16 (2.2)			11 (2.8)			5 (2.5)		5 (3.0)		1 (2.9)		
VX	1 (0.0)		1 (0.0)		0 (0.0)		1 (0.1)		0 (0.0)			0 (0.0)			0 (0.0)		0 (0.0)		0 (0.0)		
Pn-category	899		706								0.105	193									0.824
Pn0	832 (92.5)		652 (92.4)		129 (96.3)		254 (90.4)		269 (92.4)			180 (93.3)			90 (92.8)		75 (92.6)		15 (100)		
Pn1	67 (7.5)		54 (7.6)		5 (3.7)		27 (9.6)		22 (7.6)			13 (6.7)			7 (7.2)		6 (7.4)		0 (0.0)		
Grading	2.119		2.119								0.000										
G1	481 (22.7)		481 (22.7)		97 (23.7)		241 (24.0)		143 (20.3)												
G2	1.251 (59.0)		1.251 (59.0)		209 (51.0)		600 (59.6)		442 (62.9)												
G3	387 (18.3)		387 (18.3)		104 (25.4)		165 (16.4)		118 (16.8)												
pR-status	2.863		2.445								0.037	418									0.757
R0	2.818 (98.5)		2.409 (98.5)		481 (98.2)		1.167 (99.2)		761 (97.7)			409 (97.8)			200 (98.0)		174 (97.8)		35 (97.2)		
R1	27 (0.9)		23 (0.9)		5 (1.0)		7 (0.6)		11 (1.4)			4 (1.0)			2 (1.0)		2 (1.1)		0 (0.0)		
RX	18 (0.6)		13 (0.5)		4 (0.8)		2 (0.2)		7 (0.9)			5 (1.2)			2 (1.0)		2 (1.1)		1 (2.8)		

All women who had participated in QuaMaDi presented most commonly without lymph node metastases (54.1–74.5%; Table 2). Regarding the lymph node status, women in the pre-screening group were more likely to present with lymph node metastases in the non-neo group (Table 2) further substantiating that these women suffer from more aggressive tumors.

### Comparison between biopsy and resection specimens

We examined the concordances of morphology in 2.561 and grading in 983 eligible cases with corresponding biopsy and resection specimens. Regarding histological diagnoses, 2.152 (84.0%) diagnoses obtained in biopsy specimens were concordant with the diagnoses in the resection specimens. In 115 cases (4.5%), no (invasive) carcinoma was identified in the biopsy, but invasive carcinoma was found in the resection specimen (S3). Most discordant morphologies were apparent between invasive carcinoma NST and lobular carcinoma.

Likewise, an identical tumor grade was given in 760 of 983 (77.3%) corresponding biopsy and resection specimens (S4). A higher tumor grade had been documented in 40 (4.0%) biopsy specimens and a lower tumor grade in 183 (18.6%) compared with the corresponding resection specimens. Among these, two carcinomas were graded as well-differentiated carcinoma in biopsy but presented as poorly differentiated carcinoma in resection specimens. The results show that there was a tendency for undergrading in biopsies. However, overall high agreement was achieved. Tumor grading in biopsy specimens was representative in more than three quarter of the cases.

## Discussion

In this retrospective study we evaluated the histopathological characteristics and TNM classification of breast carcinoma in a large cohort of women participating in the pilot-project QuaMaDi in Germany.

Our analysis highlights several significant differences when comparing the three age groups (pre-, peri-, post-screening). In particular, over one third of breast cancers diagnosed in women younger than 50 years had the molecular subtypes triple-negative and HER2 positive. In young women these molecular subtypes were associated with more aggressive tumor biology and showed marked poorer patient prognosis compared to the other subtypes (Perou et al. 2000; Sorlie et al. 2001). Additionally, the most frequent molecular subtype was luminal B in this study (between 44.7 and 49.7%). This proportion is higher than reported in other series of breast cancer patients (range from 14 to 35%) (Sabiani et al. 2016; Collins et al. 2012).

The higher prevalence of luminal B cancers amongst this population is concordant with the greater overexpression of Ki-67 and HER2 in tumors, both of which, in the presence of ER are characteristics of luminal B cancers (Sabiani et al. 2016). Additionally, early clinical apparent cancers are more often fast-growing carcinomas and tend more likely to be detected in diagnostic breast diagnostics as QuaMaDi. Furthermore, there are marked differences in the distribution of molecular subtypes in screening programs compared to symptomatic carcinoma (Falck et al. 2016). In concordance with our data, symptomatic patients were diagnosed with more non-luminal-A tumors (Falck et al. 2016). Furthermore, tumors of elderly patients showed more ER positivity and less overexpression of HER2 and Ki-67 (Sabiani et al. 2016; Falck et al. 2016). In line with this, the incidence of triple-negative carcinomas decreased with age, as well as HER2 overexpression. The high proportion of HER2 positive and triple-negative carcinomas in women younger than 50 years indicates that QuaMaDi can identify an at risk population.

Among others the proportion of diagnosed in-situ carcinoma is used as an evaluation parameter in screening programs. According to EU guideline the proportion of in-situ carcinoma in screening programs should be above 15% (European Reference Organisation for Quality Assured Breast Screening and Diagnostic Services 2006). Although QuaMaDi is not a screening program, we achieved this goal and found in situ carcinomas in 16.7% of women < 50 years and in 15.4% of women between 50 and 69 years. Nevertheless, screening programs reach higher proportions by approximately 20% (Cooperative Association of the German Mammography Screening Program 2018; The National Health Service Breast Screening Programme 2021). The different inclusion criteria of QuaMaDi, where symptomatic women are included, may partly explain this difference. It is known that only about 20% of in-situ carcinoma become symptomatic, whereas 80% are asymptomatic and are more often detected in screening programs (Virnig et al. 2010). Furthermore, symptomatic in situ carcinomas have an increased risk of recurrence compared to lesions detected by screening (Shamliyan et al. 2010). Especially younger women (< 50 years) represent a high-risk population. In our cohort 29% of high-risk lesions and in situ carcinomas were diagnosed in younger women (< 50 years), leading to early diagnosis and identification of high-risk patients.

The majority of breast carcinoma in QuaMaDi was diagnosed at an early disease stage, i.e., 60.0–71.4% with pT1 in our cohort compared with 52% in the national epidemiological data set (Robert Koch Institute 2016). Previous studies already described the positive impact of QuaMaDi (Robert Koch Institute 2016; Obi et al. 2011). Although, length time bias can influence the distribution of tumor stage (Falck et al. 2016; Lawrence et al. 2009). Tumors diagnosed by screening programs are more often slow growing, less aggressive tumors and show more favorable tumor biology (Falck et al. 2016). The less favorable stage distribution in elderly patients is associated with fewer consultation of gynecologist for screenings or by less breast cancer awareness (Obi et al. 2011). This could lead to a delayed diagnosis of advanced disease. In addition, lead time bias must be considered. Due to the study design, length and lead time bias cannot be excluded. Nevertheless, breast carcinomas were diagnosed with less favorable and more aggressive tumor biology in our cohort. The more aggressive tumor biology, particularly in women of young disease age, could explain the lower proportion of pT1 tumors compared to women in the peri-screening age group. The overall higher proportion of pT1 tumors compared to national data indicates that QuaMaDi contributes to improved medical care for women who are unable or decline to participate in the GMSP (Obi et al. 2011; Katalinic et al. 2007).

Nodal involvement also revealed age-specific differences. We noted a decrease in nodal positive breast cancers by age 70 and an additional increase in women older than 70 years. The more frequent detection of breast carcinoma in advanced tumor stages in older women as well as the age-related decrease of immunological defense function, which favors invasion and nodal involvement are possible explanations (Daidone et al. 2003; Larbi et al. 2008; Wildiers et al. 2009). The high proportion of node negative breast carcinomas may indicate that despite the more aggressive tumor biology, breast carcinomas were diagnosed at a non-metastatic stage of disease.

Evaluating the agreement between biopsy and surgical specimen: 77.3% of cases agreed in grading and 84% were concordant in morphology. These proportions are in line to the one reported in other studies (O'Leary et al. 2004; Rakha and Ellis 2007; The Royal College of Pathologists 2016) and confirm that biopsies obtained within QuaMaDi are representative for resected specimens. The surgical materials tended to be higher grade than biopsies. Influencing factors of disagreement might be insufficient sample volume or the invasion front was not covered, so that a different value could emerge in the resected specimens (O'Leary et al. 2004). Discrepancy in morphology could be influenced by several factors: sample volume, differentiation of the tumor and regressive changes after neoadjuvant therapy. However, the influencing factors also affect screening biopsies.

Limitations of our study include its retrospective character. Furthermore, the data was based on the pathology reports of only one of four reference centers in QuaMaDi. Nevertheless, a high number of biopsies and resected specimens were included and emphasize the representativeness of our results. Missing TNM classification of all diagnosed breast carcinoma is another limitation and is due to data protection guidelines in Germany. This may have influenced the distribution of tumor and nodal stages in this study. Also, modified immunohistochemical staining protocols as well as updated international recommendations of receptor positivity or negativity are influencing factors. Although, we achieved the expected positivity rates for ER (~ 80%), PR (~ 60–70%), and HER2 (~ 15%) (Lakhani et al. 2012). Internal and external quality controls during study period ensure the representativeness and validity of the results. Nevertheless, interobserver variability cannot be excluded. But, the different evaluation algorithms affected all three age cohorts equally in the respective period.

## Conclusion

In conclusion, high-quality standards in standard care of breast diagnostics leads to an early diagnosis of breast cancer. Even if QuaMaDi cannot be compared to mammography screening, their effects on tumor stage distribution and nodal involvement are similar. Among our large cohort of women, clinicopathological features and molecular phenotypes were different across age groups. In particular, ¼ of breast cancers were diagnosed in women under 50 years and regardless of age, the tumors showed more aggressive, prognostically unfavorable tumor characteristics. Therefore, risk population could be identified. Considering additionally that 60% (Schaefer et al. 2010) of all breast cancers are diagnosed within standard care, the implementation of a high-quality breast diagnostics, as provided in QuaMaDi, can improve and complement breast cancer care.

## Supplementary Information

Below is the link to the electronic supplementary material.

Supplementary file1 (DOCX 141 kb)
