# Supplementary material for: The age-specific differences in histopathological tumor characteristics and TNM classification of breast carcinomas in Quality assured mamma diagnostic (QuaMaDi) program in the state of Schleswig–Holstein in Germany
Source: J Cancer Res Clin Oncol. 2021 Oct 27;148(2):387–400. doi: 10.1007/s00432-021-03841-x (PMC8800883; doi:10.1007/s00432-021-03841-x)
Supplement: Supplementary file 1 — Supplementary file1 (DOCX 141 kb) [file 432_2021_3841_MOESM1_ESM.docx]

**Supplemental Information**

**The age-specific differences in histopathological tumor characteristics and TNM classification of breast carcinomas in Quality assured Mamma Diagnostic (QuaMaDi) Program in the State of Schleswig-Holstein in Germany**

L.-J. Kramp,^1^ M. Mathiak,^1^ H.-M. Behrens,^1^ F. W. Schäfer^2^, M. van Mackelenbergh^2^, C. Röcken^1^

^1^ Department of Pathology, Christian-Albrechts-University, Arnold-Heller-Str. 3, Haus 14, 24105 Kiel, Germany

^2^ Department of Gynecology and Obstetrics, Campus Kiel, University Hospitals Schleswig-Holstein, Kiel

**Supplemental Data**

**S1**: B-classification [17]

| **B1**  Normal tissue or non-usable material | | - Insufficient/unusable material - Normal results with or without glandular tissue   e.g. fat tissue only, regressive changes, fibrosis |
| --- | --- | --- |
| **B2**  Benign lesions | | - Fibroadenoma, tubular adenoma - Sclerosing adenosis and - Duct ectasia - Milk duct papilloma - Pseudoangiomatous stromal hyperplasia (PASH) - Mastitis, abscess - Fat necrosis - Fibrocystic mastopathy |
| **B3**  Lesions of uncertain biological potential | | - Atypical ductal hyperplasia (ADH) or atypical epithelial proliferation of ductal type - Flat epithelial atypia - Lobular neoplasia - Phyllodes tumor - Papillary lesions - Radial scar - Mucocele-like lesions - Rare lesions e.g. adenomyoepithelioma, microglandular adenosis, spindle cell lesions |
| **B4**  Suspicious | | - Suspicion of carcinoma, but poor quality of specimen does not permit diagnosis - limited material with evidence of invasive carcinoma to perform immunohistochemical studies - atypical epithelial proliferations depending on the degree of atypia and the architecture |
| **B5** |  |  |
| Malignoma | B5a | - In situ carcinoma |
|  | B5b | - Invasive carcinoma |
|  | B5c | - Not to decide whether invasive or in situ |
|  | B5d | - Malignancy of other histogenesis or metastasis |

**S2:** Relative age distribution of 7111 histological submissions at the time of diagnosis from 01/01/2005 to 31/12/2016, color-divided by the presence or absence of malignancy. The black vertical lines mark the age groups pre-, peri- and post-screening.

**
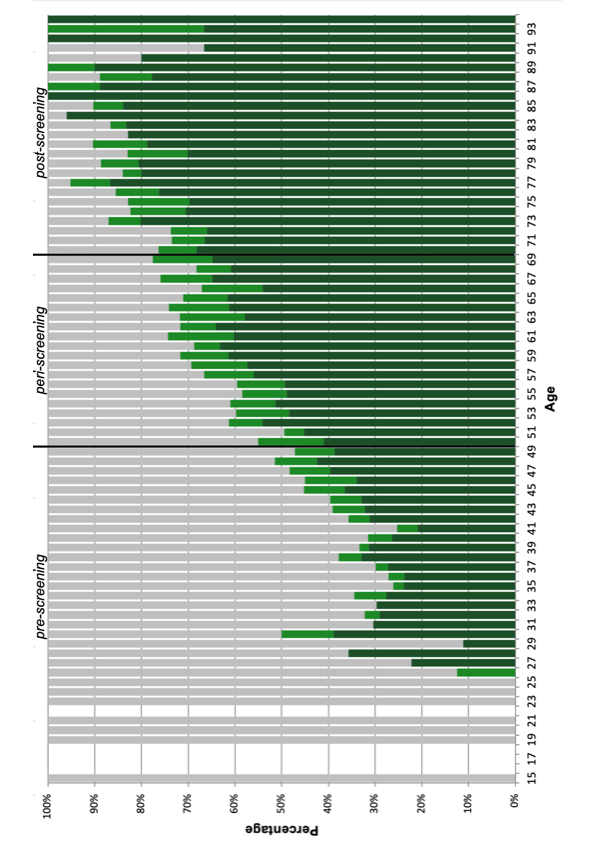
**

|  | No malignancy |
| --- | --- |
|  | Precursor lesions |
|  | Invasive carcinoma +/- precursor lesion |

**S3:** The agreement of the histologic subtypes (ICD-M) between the preoperative diagnostics (biopsy) and the resection specimens.

|  |  | **ICD-M resection specimen** | | | | | | | |  |
| --- | --- | --- | --- | --- | --- | --- | --- | --- | --- | --- |
|  |  | Invasive carcinoma not specified | Lobular carcinoma not specified | Medullary carcinoma not specified, atypical medullary carcinoma | Tubular adeno-carcinoma | Intraductal papillar adenocarci-noma with invasion | Metaplastic carcinoma  not specified | Mucinous carcinoma | Other subtypes | Total |
| **ICD-M**  **Biopsy specimen** | No diagnosis of (invasive)  carcinomas | 88 | 9 | 0 | 2 | 3 | 1 | 1 | 11 | 115 |
|  | Invasive-ductal/invasive carcinoma NST | 1677 | 58 | 28 | 14 | 3 | 3 | 4 | 31 | 1818 |
|  | Invasive-lobular carcinoma | 44 | 357 | 0 | 1 | 0 | 0 | 0 | 18 | 420 |
|  | Invasive carcinoma with medullary features/ medullary carcinoma/ atypical medullary carcinoma | 19 | 0 | 22 | 0 | 0 | 1 | 0 | 2 | 44 |
|  | Tubular carcinoma | 22 | 0 | 0 | 25 | 0 | 0 | 0 | 0 | 47 |
|  | Invasive papillary carcinoma/ solid papillary carcinoma | 10 | 0 | 4 | 0 | 8 | 1 | 0 | 4 | 27 |
|  | Metaplastic carcinoma | 0 | 0 | 0 | 0 | 0 | 4 | 0 | 2 | 6 |
|  | Mucinous carcinoma | 10 | 0 | 0 | 0 | 0 | 0 | 39 | 0 | 49 |
|  | Other subtypes | 10 | 1 | 1 | 0 | 3 | 0 | 0 | 20 | 35 |
|  | Total | 1880 | 425 | 55 | 42 | 17 | 10 | 44 | 88 | 2561 |

**S4:** The agreement of the grading between the preoperative diagnostics (Elston and Ellis grading system) and the resection specimens.

|  |  | **Resection specimen** | | |  |
| --- | --- | --- | --- | --- | --- |
|  |  | G1 | G2 | G3 | Total |
| **Pre-operative specimen** | G1 | 215 | 109 | 2 | 326 |
|  | G2 | 36 | 443 | 72 | 551 |
|  | G3 | 0 | 4 | 102 | 106 |
|  | Total | 251 | 556 | 176 | 983 |
